# Gender difference in the relationship between the ferritin and homeostasis model assessment of insulin resistance in non-diabetic Korean adults

**DOI:** 10.1371/journal.pone.0199465

**Published:** 2018-06-27

**Authors:** Hyun Yoon, Yoon Sik Kim, Jun Ho Lee, Mi Young Gi, Ju Ae Cha, Jeong Min Seong

**Affiliations:** 1 Department of Biomedical Laboratory Science, Hanlyo University, Hallyeodae-gil, Gwangyang-eup, Gwangyang-si, Jeollanam-do, South Korea; 2 Department of Biomedical Laboratory Science, Dongkang College, Dongmun-daero, Buk-gu, Gwangju, South Korea; 3 Department of Biomedical Laboratory Science, Wonkwang Health Science University, Iksan-si, South Korea; 4 Department of Nursing, Christian College of Nursing, Nam-gu, Gwangju, South Korea; 5 Department of Nursing, Chosun Nursing College, Pilmun-daero, Dong-gu, Gwangju, South Korea; 6 Department of Dental Hygiene, College of Health Science, Kangwon National University, Dogyeuhoe-ro, Dogye-eup, Samcheok-si, Gangwon-do, South Korea; National Eye Institute, UNITED STATES

## Abstract

**Background:**

The present study was conducted to assess gender difference in the relationship between the ferritin and homeostasis model assessment of insulin resistance (HOMA-IR) and beta cell function (HOMA-*B*) in non-diabetic Korean adults.

**Materials and methods:**

A sample including 5,414 adults (2,279 men, 1,529 postmenopausal women, and 1,606 premenopausal women) aged ≥ 20 years from the fifth Korean National Health and Nutrition Examination Survey (KNHANES V-1, 2010) was analyzed.

**Results:**

There were several key findings in the present study. First, in men, HOMA-IR (β = 0.119, 95% confidence interval [CI], 3.304 to 8.003) constituted the independent factor determining ferritin, but this was not the case for HOMA-*B* (β = -0.042, 95% CI, -0.100 to 0.011). Second, in postmenopausal women, HOMA-IR (β = 0.087, 95% CI, 0.899 to 5.238) was the independent factor determining ferritin, but this was not the case for HOMA-*B* (β = -0.043, 95% CI, -0.065 to 0.010). Third, in premenopausal women, neither HOMA-IR (β = -0.050, 95% CI, -3.056 to 0.364) nor HOMA-*B* (β = -0.009, 95% CI, -0.028 to 0.020) constituted the independent factors determining ferritin.

**Conclusions:**

Ferritin was positively associated with insulin resistance in non-diabetic Korean men and postmenopausal women, but not in non-diabetic Korean premenopausal women.

## Introduction

The threatening epidemic of diabetes, a disease that results from the interaction of genetic and environmental factors and that is largely driven by the increase in obesity, may be projected to affect > 400 million adults worldwide by 2030 [1, 2]. Increased insulin resistance and a decreased beta cell function are known to play a role in the pathogenesis of type 2 diabetes, and with reference to the measurements thereof, the homeostatic model assessment of insulin resistance (HOMA-IR) and the beta cell function (HOMA-*B*) are appropriate for use in large-scale epidemiological studies [3, 4].

Serum ferritin reflects the amount of iron stores in the body, since ferrous iron combined with apoferritin is stored as ferritin in many organisms [5]. Ferritin levels decrease with the onset of diseases such as iron deficiency anemia (IDA) and telogen effluvium, and increase as a result of insulin resistance, inflammation, and cardiovascular disease [6–9].

Research on ferritin and insulin resistance is being conducted all over the world. However, these relationships are not consistent across gender, ethnic groups and countries, healthy subjects, and subjects with disease (e.g., diabetes, obesity, and metabolic syndrome) [10–14]. In the non-diabetic population, the relationship between ferritin and insulin resistance may differ as a function of gender because of the difference in lifestyle, menstruation, and sex hormones [15]. Therefore, the present study aimed to investigate gender difference in the relationships between ferritin and insulin resistance and beta cell function in non-diabetic adults, using the fifth Korea National Health and Nutrition Examination Survey (KNHANES V-1) data, which is representative of the entire population of Korea.

## Materials and methods

### Study subjects

This study was based on data from the KNHANES V–1 (2010), which are the most recent data for both HOMA and ferritin levels. The KNHANES is a cross-sectional survey conducted nationwide by the Division of Korean National Health and Welfare. The KNHANES V–1 (2010) was conducted from January to December 2010. In the KNHANES V–1 (2010), 8,958 individuals over 1 year of age were sampled for the survey. Among the 6,665 subjects who participated in the KNHANES V–1, we limited the analyses to adults aged ≥ 20 years. We excluded 891 subjects whose data were lacking important analytic variables, such as the HOMA-IR, HOMA-*B*, ferritin, and various blood chemistry tests. After the exclusion of those individuals with missing values or of those who suffered from diabetes (360 subjects diagnosed with type 1 or 2 diabetes or with fasting blood glucose level ≥ 126 mg/dL), 5,414 subjects (men, 2,279; women, 3,135) were included in the statistical analysis. The KNHANES V–1 (2010) study was conducted according to the principles expressed in the Declaration of Helsinki (Institutional Review Board No, 2010-02CON-21-C). All survey participants agreed with the use of epidemiological research to identify risk factors and death causes of chronic diseases. Participants' records and information in the KNHANES were anonymous and de-identified prior to analysis. Further information can be found in “The KNHANES V–1 (2010) Sample,” which is available on the KNHANES website. The official website of KNHANES (http://knhanes.cdc.go.kr) is currently operating an English-language information homepage. The data of the respective year are available to everyone free of charge. If the applicant completes a simple subscription process and provides his/her email address on the official website of KNHANES, the data of the respective year can be downloaded free of charge. If additional information is required, the readers may contact the department responsible for the storage of data directly (Su Yeon Park, sun4070@korea.kr).

### General characteristics and blood chemistry

The research subjects were classified according to sex (men, and women), smoking habits (non-smoker or ex-smoker or current smoker), alcohol intake (yes or no), and the amount of regular exercise (yes or no). In the smoking category, participants who smoked more than one cigarette a day, those who had previously smoked but do not presently smoke, and those who never smoked were classified into the current smoker, ex-smoker, and non-smoker groups, respectively. Alcohol intake was indicated as “yes” for participants who had consumed at least one glass of alcohol every month over the past year. Regular exercise was indicated as “yes” for participants who had exercised on a regular basis (regular exercise was defined as 30 min at a time and 5 times/wk. in the case of moderate exercise, such as swimming slowly, doubles tennis, volleyball, badminton, table tennis, and carrying light objects; and for 20 min at a time and 3 times/wk. in the case of vigorous exercise, such as running, climbing, cycling fast, swimming fast, football, basketball, jump rope, squash, singles tennis, and carrying heavy objects). Anthropometric measurements included the body mass index (BMI) and waist measurement (WM), as well as measurements of the systolic blood pressure (SBP) and diastolic blood pressure (DBP). Blood chemistries included total cholesterol (TC), high density lipoprotein cholesterol (HDL-C), triglycerides (TGs), fasting blood glucose (FBG), hemoglobin (Hb), hematocrit (Hct), serum iron (Fe), total iron binding capacity (TIBC), and ferritin measurements.

### Metabolic syndrome

The metabolic syndrome (MetS) was defined using the diagnostic criteria of the National Cholesterol Education Program (NCEP) based on common clinical measures including TGs, HDL-C, blood pressure, FBG, and WM. TGs over 150 mg/dL were set as the criteria for elevated TGs. The criteria for reduced HDL-C were HDL-C of less than 50 mg/dL. FBG over 100 mg/dL was set as the criterion for elevated FBG. SBP over 130 mmHg or DBP over 85 mmHg or medication were set as the criteria for elevated blood pressure. The criteria for abdominal obesity were abdominal measurements of over 80 cm, according to the Asia-Pacific standards [16]. The MetS score indicates the presence of abdominal obesity, elevated blood pressure, elevated FBG, elevated TGs, or reduced HDL. Subjects without any of the five risk factors received an MSS 0, and those with one, two, three, and four or more of the risk factors received an MSS score of 1, 2, 3, 4, and 5, respectively [17].

### Ferritin and HOMA-IR and HOMA-*B*

The concentrations of serum ferritin were measured using an immunoturbidimetric assay (IRMA-mat Ferritin; DiaSorin, Still Water, MN, USA) using a 1470 Wizard Gamma Counter (Perkin Elmer, Turku, Finland). The quartiles of ferritin are classified as follows: in men, 1^st^ quartile, 62.94 μg/L or less; 2^nd^ quartile, 62.95–97.03 μg/L; 3^rd^ quartile, 97.04–149.28 μg/L; 4^th^ quartile, 149.29 μg/L or more. In postmenopausal women, 1^st^ quartile, 35.07 μg/L or less; 2^nd^ quartile, 35.08–54.68 μg/L; 3^rd^ quartile, 54.69–84.19 μg/L; 4^th^ quartile, 84.20 μg/L or more. In premenopausal women, 1^st^ quartile, 11.72 μg/L or less; 2^nd^ quartile, 11.73–23.84 μg/L; 3^rd^ quartile, 23.85–43.11 μg/L; 4^th^ quartile, 43.12 μg/L or more. The homeostatic model assessment of insulin resistance (HOMA-IR) and the beta cell function (HOMA-*B*) constitute a method for assessing beta cell function and insulin resistance from basal glucose and insulin concentrations. The formulas are as follows: HOMA-IR = [fasting insulin (μU/mL) × fasting plasma glucose (mg/dL)] / 405; HOMA-*B* = 360 × fasting insulin (μU/mL)/ [fasting plasma glucose (mg/dL) − 63] [18].

### Statistical analysis

The collected data were statistically analyzed using SPSS WIN version 18.0 (SPSS Inc., Chicago, IL, USA). The distributions of the participant characteristics were converted into percentages, and the successive data were presented as averages with standard deviations. The distribution and average difference in clinical characteristics according to the quartiles of ferritin were calculated using chi-square and analysis of variance (ANOVA) tests. The multiple linear regression analyses for the independent factors determining ferritin in men and pre- and postmenopausal women were adjusted for age, alcohol drinking, smoking, regular exercise, SBP, DBP, BMI, WM, TC, TGs, HDL-C, HOMA-IR, and HOMA-*B*. The significance level for all of the statistical data was set as *P* < 0.05.

## Results

### Clinical characteristics of research subjects

The clinical characteristics of the research subjects are shown in Table 1. Among the total set of subjects (5,414 subjects), in men (2,279 subjects), the serum ferritin, FBS, MetS score, HOMA-IR, and HOMA-*B* level were 113.44 ± 69.95 μg/L, 94.62 ± 9.94 mg/dL, 1.48 ± 1.27, 2.44 ± 1.51, 125.71 ± 65.79, respectively. In postmenopausal women (1,606 subjects), the serum ferritin, FBS, MetS score, HOMA-IR, and HOMA-*B* level were 64.40 ± 43.37 μg/L, 94.31 ± 10.26 mg/dL, 1.94 ± 1.33, 2.44 ± 1.23, and 129.79 ± 78.95, respectively. In premenopausal women (1,529 subjects), the serum ferritin, FBS, MetS score, HOMA-IR, and HOMA-*B* level were 30.94 ± 26.67 μg/L, 89.47 ± 8.14 mg/dL, 0.81 ± 1.02, 2.26 ± 0.99, and 146.75 ± 64.51, respectively.

**Table 1:** General characteristics.

Variables	Category	Total (n = 5,414)	Men (n = 2,279)	Postmenopausal women (n = 1,606)	Premenopausal women (n = 1,529)	*P*
Age (years)		48.43 ± 15.66	48.79 ± 15.69	60.50 ± 11.53	36.44 ± 8.29	< 0.001
	20–39 (n/%)	1,843/34.1	753/33.0	90 /5.9	1,000 /62.3	< 0.001
	40–59 (n/%)	2,096/38.7	867/38.0	623 /40.7	606 /37.7	
	≥ 60 (n/%)	1,475/27.2	659/29.0	816/55.3	0/0.0	
Alcohol drinking	Current drinker (n/%)	2,900/53.6	1,704/74.8	408/26.7	788/49.1	< 0.001
Smoking status	Current smoker (n/%)	1,143/21.1	976/42.8	68/4.4	119/7.4	< 0.001
Physical activity	Regular exerciser (n/%)	588/10.9	246/10.8	183/12.0	159/9.9	0.893
SBP (mmHg)		117.65 ± 17.46	119.83 ± 16.03	107.64 ± 13.37	124.91 ± 18.53	< 0.001
DBP (mmHg)		74.51 ± 10.48	76.94 ± 10.46	75.40 ± 10.40	70.23 ± 9.19	< 0.001
BMI (kg/m^2^)		23.50 ± 3.29	23.92 ± 3.09	23.99 ± 3.26	22.44 ± 3.36	< 0.001
WM (cm)		80.62 ± 13.81	84.44 ± 17.30	81.32 ± 9.20	74.51 ± 8.88	< 0.001
TC (mg/dL)		188.38 ± 36.02	187.74 ± 36.12	200.29 ± 36.88	177.96 ± 31.38	< 0.001
TGs (mg/dL)		127.19 ± 98.21	152.01 ± 123.69	128.73 ± 76.13	90.52 ± 55.42	< 0.001
HDL-C (mg/dL)		53.16 ± 12.78	49.68 ± 12.08	54.09 ± 12.91	57.20 ± 12.27	< 0.001
FBG (mg/dL)		93.00 ± 9.80	94.62 ± 9.94	94.31 ± 10.26	89.47 ± 8.14	< 0.001
MetS (n/%)		1,136/21.0	499/21.9	511/33.4	126/7.8	< 0.001
MetS score		1.41 ± 1.29	1.48 ± 1.27	1.94 ± 1.33	0.81 ± 1.02	< 0.001
Insulin (μU/mL)		10.28 ± 4.49	10.32 ± 5.59	10.37 ± 4.64	10.12 ± 3.97	0.311
Fe (μg/dL)		111.34 ± 47.23	126.52 ± 49.94	102.05 ± 34.43	98.63 ± 47.78	< 0.001
TIBC (μg/dL)		315.72 ± 44.82	308.52 ± 39.56	312.93 ± 43.86	328.61 ± 49.75	< 0.001
Ferritin (μg/L)		75.12 ± 63.49	113.44 ± 69.95	64.40 ± 43.37	30.94 ± 26.67	< 0.001
Hb (g/dL)		13.90 ± 1.57	15.17 ± 1.14	13.12 ± 1.05	12.82 ± 1.20	< 0.001
Hct (%)		41.28 ± 4.08	44.51 ± 3.10	39.29 ± 2.89	38.60 ± 2.97	< 0.001
HOMA-IR		2.39 ± 1.30	2.44 ± 1.51	2.44 ± 1.23	2.26 ± 0.99	< 0.001
HOMA-*B*		133.10 ± 69.98	125.71 ± 65.79	129.79 ± 78.95	146.75 ± 64.51	< 0.001

M ± SD, (n = 5,414).

SBP: systolic blood pressure, DBP: diastolic blood pressure, BMI: body mass index, WM: waist measurement, TC: total cholesterol, TGs: triglycerides, HDL-C: high density lipoprotein cholesterol, FBG: fasting blood glucose, MetS: metabolic syndrome, MetS score: metabolic syndrome score, Fe: serum iron, TIBC: total iron binding capacity, Hb: hemoglobin, Hct: hematocrit, HOMA-IR: homeostasis model assessment of insulin resistance, HOMA-*B*: homeostasis model assessment of beta-cell function.

### Clinical characteristics of subjects according to the quartiles of ferritin in men and pre- and postmenopausal women

The clinical characteristics of the research subjects according to the quartiles of ferritin in men and pre- and postmenopausal women are provided in Tables 2, 3 and 4. In men, age (*P* < 0.001), DBP (*P* = 0.003), BMI (*P* < 0.001), WM (*P* < 0.001), TC (*P* = 0.003), TGs (*P* < 0.001), FBG (*P* < 0.001), insulin (*P* < 0.001), MetS (*P* < 0.001), MetS score (*P* < 0.001), Hb (*P* < 0.001), Hct (*P* < 0.001), and HOMA-IR (*P* < 0.001) were associated with the quartiles of ferritin but SBP (*P* = 0.574), HDL-C (*P* = 0.053), HOMA-*B* (*P* = 0.152) were not (Table 2). In postmenopausal women, age (*P* < 0.001), DBP (*P* = 0.033), BMI (*P* = 0.010), WM (*P* = 0.001), TGs (*P* < 0.001), HDL-C (*P* < 0.001), FBG (*P* < 0.001), insulin (*P* = 0.001), MetS (*P* < 0.001), MetS score (*P* < 0.001), Hb (*P* < 0.001), Hct (*P* < 0.001), and HOMA-IR (*P* < 0.001) were associated with the quartiles of ferritin but SBP (*P* = 0.557), TC (*P* = 0.852), and HOMA-*B* (*P* = 0.602) were not (Table 3). In premenopausal women, age (*P* < 0.001), FBG (*P* = 0.040), Hb (*P* < 0.001), and Hct (*P* < 0.001) were associated with the quartiles of ferritin but SBP (*P* = 0.571), DBP (*P* = 0.803), BMI (*P* = 0.240), WM (*P* = 0.177), TC (*P* = 0.769), TGs (*P* = 0.147), HDL-C (*P* = 0.663), insulin (*P* = 0.341), MetS (*P* = 0.258), MetS score (*P* = 0.195), HOMA-IR (*P* = 0.164), and HOMA-*B* (*P* = 0.435) were not (Table 4).

**Table 2:** Clinical characteristics of subjects according to the ferritin quartiles in men.

Variables	Ferritin (μg/L)				*P*
	1^st^ Quartile (n = 570) (≤ 62.94)	2^nd^ Quartile (n = 570) (62.95–97.03)	3^rd^ Quartile (n = 570) (97.04–149.28)	4^th^ Quartile (n = 569) (≥ 149.29)	
Ferritin (μg/L)	41.14 ± 14.89	79.37 ± 9.82	121.78 ± 15.09	211.65 ± 54.18	< 0.001
Age (years)	51.81 ± 16.45	49.15 ± 15.27	47.11 ± 15.60	47.09 ± 14.96	< 0.001
Current drinker (n/%)	375/65.8	415/72.8	448/78.6	466/81.9	< 0.001
Current smoker (n/%)	212/37.2	226/39.6	260/45.6	278/48.9	< 0.001
Regular exerciser (n/%)	59/10.4	65/11.4	68/11.9	54/9.5	0.551
SBP (mmHg)	120.44 ± 16.61	119.23 ± 16.51	120.11 ± 15.62	119.54 ± 15.37	0.574
DBP (mmHg)	75.94 ± 10.73	76.46 ± 10.70	77.29 ± 9.96	78.07 ± 10.34	0.003
BMI (kg/m^2^)	23.27 ± 2.91	23.76 ± 3.09	24.17 ± 3.02	24.47 ± 3.23	< 0.001
WM (cm)	82.65 ± 8.62	83.64 ± 8.31	85.79 ± 8.87	85.68 ± 8.97	< 0.001
TC (mg/dL)	181.84 ± 34.07	188.16 ± 36.54	189.31 ± 34.96	191.65 ± 38.14	0.003
TGs (mg/dL)	129.61 ± 87.07	143.87 ± 135.36	158.88 ± 119.27	175.71 ± 141.36	< 0.001
HDL-C (mg/dL)	50.69 ± 12.65	49.86 ± 11.53	49.42 ± 11.45	48.76 ± 12.60	0.053
FBG (mg/dL)	93.39 ± 9.77	94.34 ± 9.16	94.94 ± 10.05	95.82 ± 10.60	< 0.001
Insulin (μU/mL)	9.68 ± 4.50	9.87 ± 3.96	10.56 ± 5.28	11.18 ± 7.74	< 0.001
MetS (n/%)	89/15.6	106/18.6	134/23.5	170/29.9	< 0.001
MetS score	1.29 ± 1.21	1.33 ± 1.89	1.55 ± 1.30	1.75 ± 1.34	< 0.001
Fe (μg/dL)	115.34 ± 48.91	127.51 ± 48.13	126.21 ± 47.79	137.06 ± 52.55	< 0.001
TIBC (μg/dL)	322.51 ± 42.89	306.11 ± 35.81	304.48 ± 37.30	300.90 ± 38.43	< 0.001
Hb	14.84 ± 1.26	15.12 ± 1.07	15.32 ± 0.99	15.42 ± 1.14	< 0.001
Hct	43.82 ± 3.37	44.33 ± 3.07	44.85 ± 2.80	45.02 ± 3.02	< 0.001
HOMA-IR	2.25 ± 1.18	2.31 ± 0.98	2.51 ± 1.48	2.69 ± 2.12	< 0.001
HOMA-*B*	123.96 ± 62.62	121.57 ± 62.93	127.48 ± 63.21	129.83 ± 73.63	0.152

M ± SD, (n = 2,279).

**Table 3:** Clinical characteristics of subjects according to the ferritin quartiles in postmenopausal women.

Variables	Ferritin (μg/L)				*P*
	1^st^ Quartile (n = 382) (≤ 35.07)	2^nd^ Quartile (n = 383) (35.08–54.68)	3^rd^ Quartile (n = 382) (54.69–84.19)	4^th^ Quartile (n = 382) (≥ 84.20)	
Ferritin (μg/L)	21.63 ± 7.36	44.94 ± 5.70	67.56 ± 8.64	123.52 ± 40.46	< 0.001
Age (years)	58.04 ± 13.30	60.32 ± 11.50	61.40 ± 10.30	62.24 ± 10.35	< 0.001
Current drinker (n/%)	73/19.1	98/25.6	125/32.7	112/29.3	< 0.001
Current smoker (n/%)	14/3.7	18/4.7	9/2.4	22/5.8	0.184
Regular exerciser (n/%)	39/10.2	50/13.2	56/14.7	38/9.9	0.130
SBP (mmHg)	124.31 ± 20.26	124.36 ± 18.23	124.95 ± 17.33	126.00 ± 18.23	0.557
DBP (mmHg)	74.33 ± 10.81	75.01 ± 10.27	75.89 ± 10.13	76.37 ± 10.29	0.033
BMI (kg/m^2^)	23.72 ± 3.19	23.72 ± 3.23	24.31 ± 3.32	24.24 ± 3.27	0.010
WM (cm)	80.72 ± 9.43	80.11 ± 8.93	82.19 ± 9.16	82.27 ± 9.14	0.001
TC (mg/dL)	199.45 ± 36.95	199.50 ± 34.94	201.38 ± 37.58	200.82 ± 38.06	0.852
TGs (mg/dL)	120.36 ± 77.09	121.47 ± 66.91	130.95 ± 78.55	142.16 ± 79.59	< 0.001
HDL-C (mg/dL)	56.43 ± 13.87	54.68 ± 12.20	53.56 ± 12.38	51.69 ± 12.70	< 0.001
FBG (mg/dL)	92.58 ± 10.39	94.42 ± 9.84	94.42 ± 10.23	95.80 ± 10.26	< 0.001
Insulin (μU/mL)	9.93 ± 4.42	10.40 ± 5.57	9.99 ± 3.66	11.16 ± 4.63	0.001
MetS (n/%)	96/25.1	127/33.2	124/32.5	164/42.9	< 0.001
MetS score	1.71 ± 1.25	1.87 ± 1.38	1.98 ± 1.33	2.22 ± 1.32	< 0.001
Fe (μg/dL)	94.44 ± 36.82	101.98 ± 31.84	104.24 ± 33.07	107.52 ± 34.59	< 0.001
TIBC (μg/dL)	341.01 ± 50.23	309.86 ± 34.71	304.36 ± 36.40	296.48 ± 38.91	< 0.001
Hb (g/dL)	12.74 ± 1.14	13.15 ± 0.98	13.23 ± 0.91	13.36 ± 1.05	< 0.001
Hct (%)	38.47 ± 3.12	39.38 ± 2.71	39.52 ± 2.56	39.77 ± 2.49	< 0.001
HOMA-IR	2.29 ± 1.20	2.46 ± 1.45	2.35 ± 0.94	2.67 ± 1.26	< 0.001
HOMA-*B*	133.26 ± 68.26	129.50 ± 105.42	125.64 ± 73.95	130.79 ± 60.80	0.602

M ± SD, (n = 1,529).

**Table 4:** Clinical characteristics of subjects according to the ferritin quartiles in premenopausal women.

Variables	Ferritin (μg/L)				*P*
	1^st^ Quartile (n = 401) (≤ 11.72)	2^nd^ Quartile (n = 402) (11.73–23.84)	3^rd^ Quartile (n = 402) (23.85–43.11)	4^th^ Quartile (n = 401) (≥ 43.12)	
Ferritin (μg/L)	6.67 ± 2.75	18.09 ± 3.44	32.61 ± 5.53	66.70 ± 27.62	< 0.001
Age (years)	37.94 ± 7.78	35.90 ± 8.51	36.61 ± 8.05	35.31 ± 8.63	< 0.001
Current drinker (n/%)	181/45.1	173/43.0	216/53.7	218/54.4	0.001
Current smoker (n/%)	15/3.7	19/4.7	29/7.2	41/10.2	< 0.001
Regular exerciser (n/%)	27/6.7	53/13.2	39/9.7	40/10.0	0.025
SBP (mmHg)	108.45 ± 13.62	107.41 ± 13.37	107.27 ± 13.65	107.42 ± 13.65	0.571
DBP (mmHg)	70.30 ± 9.10	70.30 ± 9.10	69.86 ± 9.03	70.47 ± 9.54	0.803
BMI (kg/m^2^)	22.20 ± 3.02	22.47 ± 3.31	22.42 ± 3.32	22.68 ± 3.75	0.240
WM (cm)	73.94 ± 8.13	74.25 ± 8.73	74.57 ± 9.98	75.27 ± 9.60	0.177
TC (mg/dL)	178.66 ± 29.52	178.04 ± 30.67	176.57 ± 31.60	178.56 ± 33.64	0.769
TGs (mg/dL)	85.34 ± 43.69	91.99 ± 62.25	93.99 ± 58.14	90.74 ± 55.67	0.147
HDL-C (mg/dL)	57.76 ± 12.73	57.35 ± 11.76	56.93 ± 12.39	56.77 ± 12.22	0.663
FBG (mg/dL)	90.36 ± 7.87	88.80 ± 7.62	89.13 ± 7.93	89.57 ± 9.01	0.040
Insulin (μU/mL)	10.32 ± 4.12	9.91 ± 3.75	9.98 ± 9.77	10.29 ± 4.22	0.341
MetS (n/%)	24/6.0	30/7.5	33/8.2	39/9.7	0.258
MetS score	0.78 ± 0.96	0.75 ± 1.02	0.82 ± 1.05	0.90 ± 1.05	0.195
Fe (μg/dL)	67.66 ± 44.31	106.51 ± 44.58	111.56 ± 43.61	108.74 ± 44.77	< 0.001
TIBC (μg/dL)	378.65 ± 46.15	326.05 ± 38.73	310.64 ± 35.81	299.16 ± 36.05	< 0.001
Hb (g/dL)	11.71 ± 1.52	13.04 ± 0.76	13.22 ± 0.72	13.30 ± 0.86	< 0.001
Hct (%)	36.27 ± 3.62	39.10 ± 2.15	39.49 ± 2.08	39.53 ± 2.45	< 0.001
HOMA-IR	2.32 ± 1.03	2.19 ± 0.92	2.22 ± 0.94	2.30 ± 1.06	0.164
HOMA-*B*	142.89 ± 60.67	147.25 ± 65.96	146.50 ± 63.62	150.37 ± 67.61	0.435

M ± SD, (n = 1,606).

### Multiple linear regression analyses for the independent factors determining ferritin in men and pre- and postmenopausal women

The multiple linear regression analyses for the independent factors determining ferritin in men and pre- and postmenopausal women are provided in Table 5. In men, HOMA-IR (β = 0.119, 95% confidence interval [CI], 3.304 to 8.003) was the independent factor determining ferritin, but this was not the case for HOMA-*B* (β = -0.042, 95% CI, -0.100 to 0.011). In addition, in postmenopausal women, HOMA-IR (β = 0.087, 95% CI, 0.899 to 5.238) was the independent factor determining ferritin but this was not the case for HOMA-*B* (β = -0.043, 95% CI, -0.065 to 0.010). However, in premenopausal women, neither HOMA-IR (β = -0.050, 95% CI, -3.056 to 0.364) nor HOMA-*B* (β = -0.009, 95% CI, -0.028 to 0.020) constituted the independent factors determining ferritin (Table 5).

**Table 5:** Multiple linear regression analysis for the independent factors determining ferritin by gender.

Variables	Ferritin (μg/L)								
	Men (n = 2,279)			Posmenopausal women (n = 1,529)			Premenopausal women (n = 1,606)		
	β	95% CI	*p*-value	β	95% CI	*p*-value	β	95% CI	*p*-value
Age (years)	-0.052	-0.455 –-0.015	0.037	0.138	0.290–0.749	0.004	-0.085	-0.469 –-0.089	< 0.001
Current drinker	0.091	7.896–21.578	< 0.001	0.085	3.348–13.273	0.001	0.063	0.703–6.050	0.013
Current smoker	0.032	-0.888–6.824	0.131	0.010	-3.946–5.914	0.695	0.076	1.268–6.233	0.003
Regular exerciser	-0.011	-11.580 –-6.560	0.587	-0.005	-7.290–5.934	0.841	0.006	-3.836–4.962	0.802
SBP (mmHg)	-0.067	-0.562 –-0.024	0.033	-0.105	-0.430 –-0.061	0.009	-0.109	-0.398 –-0.040	0.017
DBP (mmHg)	0.072	0.088–0.872	0.017	0.080	0.033–0.639	0.030	0.136	0.145–0.645	0.002
BMI (kg/m^2^)	0.078	0.662–2.886	0.002	0.001	-1.231–1.240	0.995	0.002	-0.767–0.800	0.967
WM (cm)	0.015	-0.117–0.239	0.499	0.002	-0.432–0.452	0.966	0.064	-0.108–0.496	0.208
TC (mg/dL)	0.025	-0.038–0.136	0.266	0.020	-0.043–0.089	0.489	0.045	-0.011–0.088	0.130
TGs (mg/dL)	0.082	0.019–0.073	0.001	0.057	-0.001–0.067	0.059	-0.008	-0.032–0.024	0.788
HDL-C (mg/dL)	-0.018	-0.373–0.163	0.442	-0.072	-0.455 –-0.037	0.020	-0.072	-0.285 –-0.028	0.017
HOMA-IR	0.119	3.304–8.003	< 0.001	0.087	0.899–5.238	0.006	-0.050	-3.056–0.364	0.123
HOMA-*B*	-0.042	-0.100–0.011	0.115	-0.043	-0.065–0.010	0.147	-0.009	-0.028–0.020	0.760

(*n* = 5,414).

## Discussion

The present study investigates the association between ferritin and insulin resistance in non-diabetic Korean adults. The key finding of the present study was that ferritin was positively associated with HOMA-IR levels in men and postmenopausal women but not in premenopausal women.

Ferritin, which is an iron storage protein, is found as ferrous iron combined with apoferritin in every cell, such as in the liver, spleen, heart, and kidneys, and its concentration varies according to gender and age [19]. Serum ferritin is widely recognized as an acute phase reactant and marker of acute and chronic inflammation, and is nonspecifically elevated in a wide range of inflammatory conditions including diabetes, MetS, chronic kidney disease, and autoimmune disorders [20–23].

Research on the association between ferritin and insulin resistance in the non-diabetic population is being conducted all over the world. Moutinho Monteiro and colleagues reported that ferritin was associated with HOMA-IR in non-diabetic Brazilians (*P* = 0.026) [24] and Li and colleagues reported that ferritin was associated with HOMA-IR in non-diabetic elderly Chinese (*P* < 0.001) [25]. However, some studies have reported that ferritin was not associated with insulin resistance in the non-diabetic population. Zafar and colleagues reported that ferritin was associated with HOMA-IR in both the non-diabetic offspring of T2DM parents (*P* = 0.463) and the non-diabetic offspring of non-diabetic parents (*P* = 0.628) [26]. Furthermore, Chen and colleagues reported that ferritin was associated with elevated HOMA-IR in non-diabetic US adults in non-adjustment (*P* < 0.001). However, the significance of these results disappeared in the case of multivariate adjustment (*P* = 0.052) [27]. These inconsistent results may be due to differences in race, country, and lifestyle. In particular, ferritin levels vary considerably between genders because of poor diet and menstrual blood loss [28] and the relationship between ferritin and insulin resistance may differ by gender.

As described above, reduced serum ferritin is a valuable clinical marker for the diagnosis and treatment of IDA; in contrast, elevated serum ferritin is widely recognized as an acute phase reactant and marker of both acute and chronic inflammation. However, with reference to the serum ferritin level according to gender in non-diabetic populations, it remains unclear whether the level of ferritin has a positive effect on IDA and whether any level of ferritin has a negative effect on the creation of insulin resistance. Currently, research on the association between ferritin and insulin resistance by gender in the non-diabetic population is scarce. Bonfils and colleagues reported that ferritin was associated with HOMA-IR in Danish men (*P* < 0.001), but not in Danish women (*P* = 0.89) [29]. They suggested that there may be a threshold of serum ferritin of 52 μg/L, above which elevated serum ferritin levels are associated with surrogate insulin resistance measures. Some studies have suggested that a serum ferritin level higher than 70 μg/L in general populations is more appropriate for use as an acute and chronic inflammatory marker [30, 31]. In contrast, Sheu and colleagues reported that ferritin was associated with HOMA-IR in Chinese women (*P* = 0.003), but not in men (*P* = 0.424) [32]. In addition, Jehn and colleagues reported that ferritin was associated with HOMA-IR in US men (*P* = 0.04) and postmenopausal women (*P* < 0.001), but not in premenopausal women (*P* = 0.98) [33]. In the present study, serum ferritin was positively associated with Hb in men and post- and premenopausal women. In terms of IDA, this may be a positive effect for the prevention of IDA in both men and women. However, serum ferritin was positively associated with HOMA-IR in men and postmenopausal women but not in premenopausal women, and our results were similar to the findings of Jehn and colleagues [33]. In our study, there were interesting results in relation to gender difference in the association between ferritin and the insulin resistance–related indices in the non-diabetic populations (Supplement 1). Based on the theoretical background of a previous study [29], we believe that a threshold value of the serum ferritin level in non-diabetic populations is reasonable enough in the case of insulin resistance. Among the quartiles of serum ferritin in non-diabetic premenopausal women, the serum ferritin level (M ± SD) in all quartiles was lower than the threshold of serum ferritin (52 μg/L) suggested by the study of Bonfils and colleagues. Therefore, serum ferritin was not associated with HOMA-IR or with insulin resistance–related indices, such as FBG and the MetS score. However, among the quartiles of serum ferritin in non-diabetic men, the serum ferritin level (M ± SD) in the 2^nd^, 3^rd^, and 4^th^ quartile was higher than the threshold of serum ferritin (52 μg/L) suggested by the study of Bonfils and colleagues. In addition, among the quartiles of serum ferritin in non-diabetic postmenopausal women, the serum ferritin level (M ± SD) in the 3^rd^ and 4^th^ quartile was higher than the threshold of serum ferritin (52 μg/L) suggested by the study of Bonfils and colleagues. Therefore, serum ferritin was positively associated with HOMA-IR as well as with insulin resistance–related indices, such as FBG and the MetS score.

On the other hand, we think that the gender difference in the relationship between ferritin and insulin resistance is caused by sexual hormones, such as estrogen. Estrogen deficiency in postmenopausal women has been well established as the main causative factor in menopausal symptoms and diseases. The levels of serum ferritin are increased two- to threefold from before menopause to after menopause and an increased percentage of iron as a result of menopause could be a risk factor affecting the health of postmenopausal women [34]. Estrogen, and the estrogen receptor alpha (ER-α) in particular, are associated with glucose homeostasis. Heine and colleagues reported that ER-α knock-out mice exhibited insulin resistance and glucose intolerance [35]. Meyer and colleagues suggested that estrogen therapy in men is also meaningful in the treatment of insulin resistance [36]. The relationship between ferritin and insulin resistance is still unclear. However, there are several possible explanations for the relationship between serum ferritin and insulin resistance. First, hepcidin reduces the absorption of iron from the intestine, while inhibiting iron egress from macrophages [37]. Increased hepcidin expression causes iron deficiency anemia and decreased hepcidin expression leads to iron overload in the body. Insulin resistance is associated with reduced hepcidin synthesis and it may lead to increased body iron stores in insulin resistant cases [38]. Second, ferritin, which is an inflammatory factor, has been associated with the tumor necrosis factor-α (TNF-α) and interleukin-6 (IL-6), while elevated TNF-α and IL-6 may contribute to insulin resistance [39–41]. Third, since iron itself can form highly reactive free radicals, iron can interfere with glucose metabolism and induce insulin resistance [42, 43].

The present study has some limitations. First, KNHANES data are representative national data in Korea. However, considering the sample size and study population, this study imposes constraints on generalization. Second, hepcidin and the C reactive protein (CRP) constitute important determinants of the serum ferritin levels. However, hepcidin and CRP were not employed in the KNHANES V-1 study. The serum ferritin levels for CRP and hepcidin should be included as variables for ferritin in future studies. Third, because this study was a cross-sectional study, the ability to establish a causal relationship between ferritin and insulin resistance was limited. Therefore, more accurate results might be obtained by conducting a cohort study.

## Conclusions

We assessed the gender differences in the relationship between ferritin and insulin resistance in non-diabetic Korean adults. Ferritin was positively associated with HOMA-IR levels in non-diabetic Korean men and postmenopausal women but not in non-diabetic Korean premenopausal women.

## Supporting information

S1 Table Comparisons of hemoglobin, FBG, and MetS score according to the ferritin quartiles by gender. (DOCX)
