# Supplementary material for: Gender difference in the relationship between the ferritin and homeostasis model assessment of insulin resistance in non-diabetic Korean adults
Source: PLoS One. 2018 Jun 27;13(6):e0199465. doi: 10.1371/journal.pone.0199465 (PMC6021102; doi:10.1371/journal.pone.0199465)
Supplement: S1 Table — (DOCX) [file pone.0199465.s001.docx]

**Supplement 1** Comparisons of hemoglobin, FBG, and MetS score according to the ferritin quartiles by gender

(n = 5,414)

| Gender | Category | Hemoglobin (g/dL) ^*^ | FBG (mg/dL) ^*^ | MetS score ^*^ |
| --- | --- | --- | --- | --- |
| Men (n = 2,279) |  |  |  |  |
| Ferritin | 1^st^ Quartile | 14.99 ± 0.04 (14.91–15.08) | 93.54 ± 0.38 (92.79–94.29) | 1.40 ± 0.04 (1.33–1.47) |
|  | 2^nd^ Quartile | 15.15 ± 0.04 (15.06–15.23) | 94.49 ± 0.38 (93.75–95.22) | 1.42 ± 0.04 (1.36–1.50) |
|  | 3^rd^ Quartile | 15.26 ± 0.04 (15.18–15.35) | 94.94 ± 0.38 (94.19–95.68) | 1.50 ± 0.04 (1.43–1.57) |
|  | 4^th^ Quartile | 15.29 ± 0.04 (15.20–15.37) | 95.47 ± 0.38 (94.73–96.22) | 1.61 ± 0.04 (1.54–1.67) |
| *P* |  | < 0.001 | 0.004 | < 0.001 |
| Postmenopausal women (n = 1,606) |  |  |  |  |
| Ferritin | 1^st^ Quartile | 12.76 ± 0.05 (12.66–12.86) | 93.00 ± 0.50 (92.02–93.98) | 1.87 ± 0.04 (1.80–1.95) |
|  | 2^nd^ Quartile | 13.19 ± 0.05 (13.09–13.28) | 94.81 ± 0.49 (93.84–95.78) | 1.90 ± 0.04 (1.83–1.98) |
|  | 3^rd^ Quartile | 13.20 ± 0.05 (13.10–13.30) | 94.06 ± 0.49 (93.10–95.03) | 2.00 ± 0.04 (1.93–2.08) |
|  | 4^th^ Quartile | 13.33 ± 0.05 (13.23–13.43) | 95.21 ± 0.49 (94.24–96.18) | 2.01 ± 0.04 (1.93–2.08) |
| *P* |  | < 0.001 | 0.010 | 0.031 |
| Premenopausal women (n = 1,529) |  |  |  |  |
| Ferritin | 1^st^ Quartile | 11.74 ± 0.05 (11.64–11.84) | 90.20 ± 0.38 (89.46–90.94) | 0.83 ± 0.03 (0.77–0.89) |
|  | 2^nd^ Quartile | 13.03 ± 0.05 (12.93–13.13) | 88.96 ± 0.38 (88.22–89.70) | 0.76 ± 0.03 (0.70–0.82) |
|  | 3^rd^ Quartile | 13.23 ± 0.05 (13.14–13.33) | 89.13 ± 0.38 (88.40–89.86) | 0.81 ± 0.03 (0.75–0.87) |
|  | 4^th^ Quartile | 13.27 ± 0.05 (13.17–13.37) | 89.62 ± 0.38 (88.88–90.36) | 0.86 ± 0.03 (0.80–0.92) |
| *P* |  | < 0.001 | 0.087 | 0.112 |

^*^ Adjusted for age, **alcohol drinking**, smoking, **regular exercise**, SBP, DBP, BMI, WM, TC, TGs, and HDL-C.
